# 2-(1*H*-Benzotriazol-1-yl)acetohydrazide

**DOI:** 10.1107/S1600536809021199

**Published:** 2009-06-10

**Authors:** Yan-Xia Zhang, Zhi-Qiang Shi

**Affiliations:** a Department of Materials Science and Chemical Engineering, Taishan University, 271021 Taian, Shandong, People’s Republic of China

## Abstract

The title compound, C_8_H_9_N_5_O, was synthesized by the reaction of ethyl 2-(benzotriazol-1-yl)acetate with hydrazine hydrate in ethanol. In the amide group, the C—N bond is relatively short [1.3283 (16) Å], suggesting some degree of electronic delocalization in the mol­ecule. In the crystal structure, mol­ecules are linked into infinite chains along the *a* axis by inter­molecular O—H⋯N hydrogen bonding.

## Related literature

For general background to multiple-hydrogen-bonding *N*-heterocyclic systems as potential supra­molecular reagents, see: Portalone (2007 ▶); Portalone & Colapietro (2007 ▶, 2008 ▶); For related structures, see: Shi *et al.* (2007*a* ▶,*b* ▶); Ji *et al.* (2008 ▶); For bond-length data, see: Allen *et al.* (1987 ▶). 
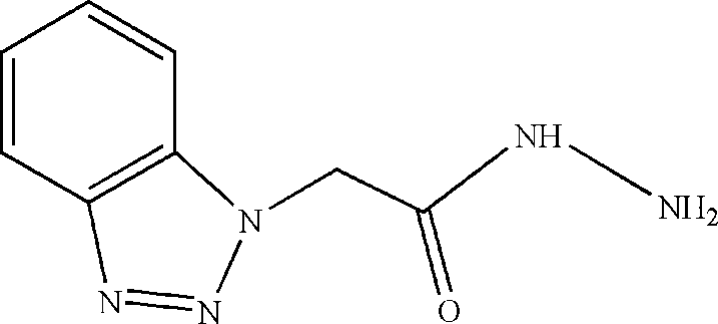


## Experimental

### 

#### Crystal data

C_8_H_9_N_5_O
*M* *_r_* = 191.20
Monoclinic, 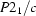

*a* = 5.1434 (9) Å
*b* = 6.5885 (12) Å
*c* = 25.754 (5) Å
β = 94.227 (3)°
*V* = 870.4 (3) Å^3^
*Z* = 4
Mo *K*α radiation
μ = 0.11 mm^−1^
*T* = 295 K
0.12 × 0.10 × 0.06 mm

#### Data collection

Bruker APEXII CCD area-detector diffractometer
Absorption correction: multi-scan (*SADABS*; Bruker, 2005 ▶) *T*_min_ = 0.988, *T*_max_ = 0.994
4367 measured reflections
1528 independent reflections
1364 reflections with *I* > 2σ(*I*)
*R*_int_ = 0.015

#### Refinement

*R*[*F* ^2^ > 2σ(*F* ^2^)] = 0.030
*wR*(*F* ^2^) = 0.079
*S* = 1.04
1528 reflections
135 parameters
H atoms treated by a mixture of independent and constrained refinement
Δρ_max_ = 0.13 e Å^−3^
Δρ_min_ = −0.15 e Å^−3^

### 

Data collection: *APEX2* (Bruker, 2005 ▶); cell refinement: *SAINT* (Bruker, 2005 ▶); data reduction: *SAINT*; program(s) used to solve structure: *SHELXTL* (Sheldrick, 2008 ▶); program(s) used to refine structure: *SHELXTL*; molecular graphics: *SHELXTL*; software used to prepare material for publication: *SHELXTL*.

## Supplementary Material

Crystal structure: contains datablocks global, I. DOI: 10.1107/S1600536809021199/bg2267sup1.cif

Structure factors: contains datablocks I. DOI: 10.1107/S1600536809021199/bg2267Isup2.hkl

Additional supplementary materials:  crystallographic information; 3D view; checkCIF report

## Figures and Tables

**Table 1:** Hydrogen-bond geometry (Å, °)

*D*—H⋯*A*	*D*—H	H⋯*A*	*D*⋯*A*	*D*—H⋯*A*
N4—H1⋯O1^i^	0.86	2.18	2.9977 (14)	159

Symmetry code: (i) 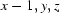.

**Fig. 1.**
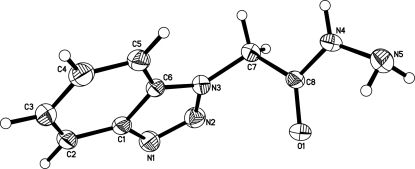
The molecular structure of (I), with displacement ellipsoids drawn at the 30% probability level.

**Fig. 2.**
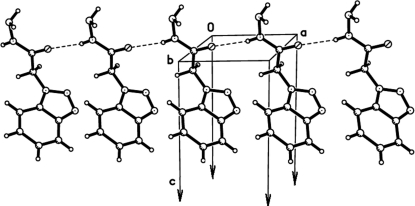
The structure of the infinite chains formed via hydrogen bonds, H atoms have been omitted for clarity. The dashed lines indicate hydrogen bonds.

## supplementary crystallographic information

### Comment

In our previous papers(Shi *et al.*, 2007a; Shi *et al.*, 2007b; Ji *et al.*, 2008;), we have reported a number of Schiff-bases by the reaction of benzotriazol-1-yl-acetic acid hydrazide with relevant aldehyde or ketone. As a part of a more general study of multiple-hydrogen-bonding N-heterocyclic systems as potential supramolecular reagents (Portalone, 2007; Portalone & Colapietro, 2007, 2008), the title compound, (I), was synthesized and its crystal structure determined. The asymmetric unit of the (I) comprises one independent molecule. In (I) (Fig. 1), the bond lengths and angles are in good agreement with the expected values (Allen *et al.*, 1987). In the crystal structure (Fig. 2), the molecules are linked into infinite chains by O—H···N hydrogen bond.

### Experimental

The title compound was synthesized by the reaction of benzotriazol-1-yl-acetic acid ethyl ester(1 mmol) with hydrazine hydrate 85% (1.1 mmol)in ethanol (20 ml) under reflux conditions (348 K) for 24 h. The solvent was removed and the solid product recrystallized from tetrahydrofuran. After four days colorless crystals suitable for X-ray diffraction study were obtained.

### Refinement

All H atoms were placed in idealized positions (C—H = 0.93— 0.97 Å, N—H = 0.86 Å) and refined as riding atoms. For those bound to C, *U*_iso_(H) = 1.2 or 1.5*U*_eq_(C). while for those bound to N, *U*_iso_(H) = 1.2 *U*_eq_(N).

### Crystal data

**Table:**

C_8_H_9_N_5_O	*F*(000) = 400
*M**_r_* = 191.20	*D*_x_ = 1.459 Mg m^−^^3^
Monoclinic, *P*2_1_/*c*	Mo *K*α radiation, λ = 0.71073 Å
Hall symbol: -P 2ybc	Cell parameters from 2593 reflections
*a* = 5.1434 (9) Å	θ = 2.4–28.0°
*b* = 6.5885 (12) Å	µ = 0.11 mm^−^^1^
*c* = 25.754 (5) Å	*T* = 295 K
β = 94.227 (3)°	Block, colorless
*V* = 870.4 (3) Å^3^	0.12 × 0.10 × 0.06 mm
*Z* = 4	

### Data collection

**Table:**

Bruker APEXII CCD area-detector diffractometer	1528 independent reflections
Radiation source: fine-focus sealed tube	1364 reflections with *I* > 2σ(*I*)
graphite	*R*_int_ = 0.015
φ and ω scans	θ_max_ = 25.1°, θ_min_ = 3.2°
Absorption correction: multi-scan (*SADABS*; Bruker, 2005)	*h* = −4→6
*T*_min_ = 0.988, *T*_max_ = 0.994	*k* = −7→7
4367 measured reflections	*l* = −28→30

### Refinement

**Table:**

Refinement on *F*^2^	Primary atom site location: structure-invariant direct methods
Least-squares matrix: full	Secondary atom site location: difference Fourier map
*R*[*F*^2^ > 2σ(*F*^2^)] = 0.030	Hydrogen site location: inferred from neighbouring sites
*wR*(*F*^2^) = 0.079	H atoms treated by a mixture of independent and constrained refinement
*S* = 1.04	*w* = 1/[σ^2^(*F*_o_^2^) + (0.0333*P*)^2^ + 0.233*P*] where *P* = (*F*_o_^2^ + 2*F*_c_^2^)/3
1528 reflections	(Δ/σ)_max_ < 0.001
135 parameters	Δρ_max_ = 0.13 e Å^−^^3^
0 restraints	Δρ_min_ = −0.15 e Å^−^^3^

### Special details

**Table:**

Geometry. All e.s.d.'s (except the e.s.d. in the dihedral angle between two l.s. planes) are estimated using the full covariance matrix. The cell e.s.d.'s are taken into account individually in the estimation of e.s.d.'s in distances, angles and torsion angles; correlations between e.s.d.'s in cell parameters are only used when they are defined by crystal symmetry. An approximate (isotropic) treatment of cell e.s.d.'s is used for estimating e.s.d.'s involving l.s. planes.
Refinement. Refinement of *F*^2^ against ALL reflections. The weighted *R*-factor *wR* and goodness of fit *S* are based on *F*^2^, conventional *R*-factors *R* are based on *F*, with *F* set to zero for negative *F*^2^. The threshold expression of *F*^2^ > σ(*F*^2^) is used only for calculating *R*-factors(gt) *etc*. and is not relevant to the choice of reflections for refinement. *R*-factors based on *F*^2^ are statistically about twice as large as those based on *F*, and *R*- factors based on ALL data will be even larger.

### Fractional atomic coordinates and isotropic or equivalent isotropic displacement parameters (Å^2^)

**Table:**

	*x*	*y*	*z*	*U*_iso_*/*U*_eq_	
O1	1.08675 (16)	0.24854 (14)	0.01128 (4)	0.0432 (3)	
N1	1.3431 (2)	0.14371 (19)	0.16751 (5)	0.0505 (3)	
N2	1.2038 (2)	0.05513 (19)	0.12958 (5)	0.0496 (3)	
N3	0.9953 (2)	0.17411 (18)	0.11547 (4)	0.0422 (3)	
N4	0.65313 (19)	0.25384 (17)	−0.00905 (4)	0.0419 (3)	
H1	0.5024	0.2240	0.0012	0.050*	
N5	0.6667 (2)	0.3428 (2)	−0.05866 (5)	0.0503 (3)	
C1	1.2246 (2)	0.3253 (2)	0.17817 (5)	0.0406 (3)	
C2	1.2914 (3)	0.4759 (2)	0.21540 (5)	0.0496 (4)	
H2	1.4385	0.4637	0.2384	0.060*	
C3	1.1312 (3)	0.6408 (2)	0.21632 (6)	0.0537 (4)	
H3	1.1698	0.7424	0.2407	0.064*	
C4	0.9094 (3)	0.6612 (2)	0.18138 (6)	0.0534 (4)	
H4	0.8067	0.7768	0.1832	0.064*	
C5	0.8397 (3)	0.5166 (2)	0.14492 (5)	0.0466 (3)	
H5	0.6933	0.5306	0.1218	0.056*	
C6	1.0018 (2)	0.3468 (2)	0.14460 (5)	0.0377 (3)	
C7	0.8079 (3)	0.1139 (2)	0.07360 (5)	0.0460 (3)	
H7A	0.6341	0.1507	0.0825	0.055*	
H7B	0.8133	−0.0323	0.0695	0.055*	
C8	0.8630 (2)	0.21429 (19)	0.02251 (5)	0.0356 (3)	
H9	0.723 (3)	0.250 (2)	−0.0799 (6)	0.073 (6)*	
H8	0.785 (3)	0.441 (2)	−0.0551 (8)	0.078 (6)*	

### Atomic displacement parameters (Å^2^)

**Table:**

	*U*^11^	*U*^22^	*U*^33^	*U*^12^	*U*^13^	*U*^23^
O1	0.0277 (5)	0.0555 (6)	0.0466 (5)	−0.0038 (4)	0.0045 (4)	−0.0023 (4)
N1	0.0450 (7)	0.0599 (8)	0.0460 (7)	0.0030 (6)	−0.0010 (5)	0.0029 (6)
N2	0.0472 (7)	0.0518 (7)	0.0500 (7)	0.0030 (6)	0.0051 (5)	0.0005 (6)
N3	0.0399 (6)	0.0508 (7)	0.0358 (6)	−0.0038 (5)	0.0024 (5)	−0.0031 (5)
N4	0.0272 (5)	0.0526 (7)	0.0457 (6)	−0.0030 (5)	0.0021 (4)	−0.0043 (5)
N5	0.0434 (7)	0.0582 (8)	0.0483 (7)	0.0013 (6)	−0.0040 (6)	0.0003 (6)
C1	0.0351 (7)	0.0530 (8)	0.0342 (7)	−0.0047 (6)	0.0054 (5)	0.0039 (6)
C2	0.0394 (7)	0.0720 (10)	0.0372 (7)	−0.0136 (7)	0.0009 (6)	−0.0029 (7)
C3	0.0515 (9)	0.0611 (10)	0.0497 (9)	−0.0159 (7)	0.0114 (7)	−0.0143 (7)
C4	0.0499 (9)	0.0521 (9)	0.0598 (9)	−0.0013 (7)	0.0145 (7)	−0.0043 (7)
C5	0.0382 (7)	0.0574 (9)	0.0441 (8)	−0.0003 (6)	0.0025 (6)	0.0020 (7)
C6	0.0346 (7)	0.0484 (8)	0.0303 (6)	−0.0061 (6)	0.0053 (5)	0.0010 (6)
C7	0.0387 (7)	0.0582 (9)	0.0412 (7)	−0.0125 (6)	0.0044 (6)	−0.0072 (6)
C8	0.0292 (6)	0.0384 (7)	0.0392 (7)	−0.0035 (5)	0.0027 (5)	−0.0110 (5)

### Geometric parameters (Å, °)

**Table:**

O1—C8	1.2280 (14)	C1—C2	1.405 (2)
N1—N2	1.3057 (16)	C2—C3	1.365 (2)
N1—C1	1.3795 (19)	C2—H2	0.9300
N2—N3	1.3559 (16)	C3—C4	1.407 (2)
N3—C6	1.3620 (17)	C3—H3	0.9300
N3—C7	1.4478 (16)	C4—C5	1.367 (2)
N4—C8	1.3283 (16)	C4—H4	0.9300
N4—N5	1.4120 (17)	C5—C6	1.3957 (19)
N4—H1	0.8600	C5—H5	0.9300
N5—H9	0.884 (9)	C7—C8	1.5179 (18)
N5—H8	0.888 (9)	C7—H7A	0.9700
C1—C6	1.3909 (18)	C7—H7B	0.9700
			
N2—N1—C1	108.09 (11)	C4—C3—H3	119.1
N1—N2—N3	108.75 (11)	C5—C4—C3	122.15 (14)
N2—N3—C6	110.41 (10)	C5—C4—H4	118.9
N2—N3—C7	120.74 (12)	C3—C4—H4	118.9
C6—N3—C7	128.83 (12)	C4—C5—C6	115.87 (13)
C8—N4—N5	122.88 (10)	C4—C5—H5	122.1
C8—N4—H1	118.6	C6—C5—H5	122.1
N5—N4—H1	118.6	N3—C6—C1	104.05 (11)
N4—N5—H9	108.2 (12)	N3—C6—C5	133.05 (12)
N4—N5—H8	106.8 (13)	C1—C6—C5	122.90 (12)
H9—N5—H8	108.4 (17)	N3—C7—C8	111.69 (10)
N1—C1—C6	108.69 (12)	N3—C7—H7A	109.3
N1—C1—C2	131.22 (13)	C8—C7—H7A	109.3
C6—C1—C2	120.08 (13)	N3—C7—H7B	109.3
C3—C2—C1	117.16 (13)	C8—C7—H7B	109.3
C3—C2—H2	121.4	H7A—C7—H7B	107.9
C1—C2—H2	121.4	O1—C8—N4	123.60 (12)
C2—C3—C4	121.83 (14)	O1—C8—C7	121.49 (11)
C2—C3—H3	119.1	N4—C8—C7	114.87 (10)
			
C1—N1—N2—N3	−0.50 (14)	C7—N3—C6—C5	0.5 (2)
N1—N2—N3—C6	0.98 (15)	N1—C1—C6—N3	0.71 (13)
N1—N2—N3—C7	179.30 (11)	C2—C1—C6—N3	−178.58 (12)
N2—N1—C1—C6	−0.15 (14)	N1—C1—C6—C5	−179.03 (12)
N2—N1—C1—C2	179.04 (14)	C2—C1—C6—C5	1.68 (19)
N1—C1—C2—C3	−179.77 (14)	C4—C5—C6—N3	178.92 (13)
C6—C1—C2—C3	−0.66 (19)	C4—C5—C6—C1	−1.43 (19)
C1—C2—C3—C4	−0.5 (2)	N2—N3—C7—C8	−97.25 (14)
C2—C3—C4—C5	0.7 (2)	C6—N3—C7—C8	80.72 (16)
C3—C4—C5—C6	0.2 (2)	N5—N4—C8—O1	−1.0 (2)
N2—N3—C6—C1	−1.02 (13)	N5—N4—C8—C7	−178.60 (12)
C7—N3—C6—C1	−179.17 (12)	N3—C7—C8—O1	34.61 (18)
N2—N3—C6—C5	178.67 (14)	N3—C7—C8—N4	−147.76 (12)

### Hydrogen-bond geometry (Å, °)

**Table:**

*D*—H···*A*	*D*—H	H···*A*	*D*···*A*	*D*—H···*A*
N4—H1···O1^i^	0.86	2.18	2.9977 (14)	159

Symmetry codes: (i) *x*−1, *y*, *z*.
